# On Detecting Cryptojacking on Websites: Revisiting the Use of Classifiers

**DOI:** 10.3390/s22239219

**Published:** 2022-11-27

**Authors:** Fredy Andrés Aponte-Novoa, Daniel Povedano Álvarez, Ricardo Villanueva-Polanco, Ana Lucila Sandoval Orozco, Luis Javier García Villalba

**Affiliations:** 1 Department of Computer Science and Engineering, Universidad del Norte, Barranquilla 081007, Colombia; 2 Department of Systems Engineering, Universidad Santo Tomás, Tunja 150003, Colombia; 3 Group of Analysis, Security and Systems (GASS), Department of Software Engineering and Artificial Intelligence (DISIA), Faculty of Computer Science and Engineering, Office 431, Universidad Complutense de Madrid (UCM), Calle Profesor José García Santesmases 9, Ciudad Universitaria, 28040 Madrid, Spain

**Keywords:** blockchain, cryptojacking, illegal mining, malware, machine learning

## Abstract

Cryptojacking or illegal mining is a form of malware that hides in the victim’s computer and takes the computational resources to extract cryptocurrencies in favor of the attacker. It generates significant computational consumption, reducing the computational efficiency of the victim’s computer. This attack has increased due to the rise of cryptocurrencies and their profitability and its difficult detection by the user. The identification and blocking of this type of malware have become an aspect of research related to cryptocurrencies and blockchain technology; in the literature, some machine learning and deep learning techniques are presented, but they are still susceptible to improvement. In this work, we explore multiple Machine Learning classification models for detecting cryptojacking on websites, such as Logistic Regression, Decision Tree, Random Forest, Gradient Boosting Classifier, *k*-Nearest Neighbor, and XGBoost. To this end, we make use of a dataset, composed of network and host features’ samples, to which we apply various feature selection methods such as those based on statistical methods, e.g., Test Anova, and other methods as Wrappers, not only to reduce the complexity of the built models but also to discover the features with the greatest predictive power. Our results suggest that simple models such as Logistic Regression, Decision Tree, Random Forest, Gradient Boosting, and *k*-Nearest Neighbor models, can achieve success rate similar to or greater than that of advanced algorithms such as XGBoost and even those of other works based on Deep Learning.

## 1. Introduction

Cryptojacking is an illegal and unauthorized mining activity on the victim’s computer, using the computational power of the victim’s computer to extract cryptocurrencies, which generates large computational consumption, reducing the computational efficiency of the victim’s computer. Moreover, this attack may be used by a powerful attacker to increment their computationally power, posing a risk to any blockchain based on mining [1,2,3,4,5,6,7].

Cryptojacking detection techniques such as browser extensions and antiviruses provide a partial solution to the cryptojacking problem since attackers can avoid them by employing obfuscation techniques or renewing domains or malicious scripts relatively frequently [8]. Cryptojacking boomed with the birth of service providers that offer ready-to-use implementations of mining scripts in web browsers. Therefore, attackers can reach many more victims via websites. These service providers are coinhive [9] and cryptoloot [10].

Cryptojacking on websites uses JavaScript code to mine cryptocurrencies. This technique does not require installing javascript code to perform the mining process. All it takes is for the user to load the infected website in their browser for the illegal mining code to execute in the browser of the victim’s computer [11]. According to [12], many websites have been infected by cryptojacking, such as personal blogs up to Alexa-ranking websites. Moreover, it noted that as of January 2022, there were around 3000 websites that offered online cryptojacking scripts.

As evidenced in [13,14,15,16,17,18,19], there is a recent trend of applying specialized deep learning models to detect cryptojacking in websites. However, specialized deep learning models might pose challenges with reference to their deployment and performance. Thus, this paper seeks to explore machine learning models (comparatively simpler than deep learning models) for cryptojacking classification to identify which of these machine learning models may render desirable results. Hence, this work takes previous works as a reference, particularly the recent specializaed deep learning model and the dataset presented and collected in  [14]. We use this dataset to train and validate multiple machine learning classification algorithms to detect cryptojacking on websites and make a comparison between the machine learning models and the specialized deep learning model.

The rest of this paper is structured as follows: In Section 2, we present three cases of cryptojacking as a motivation for addressing cryptojacking detection. Section 3 presents related works on cryptojacking detection techniques. Section 4 describes the methodology we follow for our study. Also, we describe the dataset’s selection and content and present an exploratory data analysis. This section also shows data correlation and clustering, as well as the feature selection, split, and normalization process. At the end of this section, we present some machine-learning models for detecting cryptojacking on websites. Section 5 analyses the results we obtained after going through our methodology. Section 6 narrates a possible integration scenario of our techniques with other known approaches for detecting malware and filtering/blocking websites. Finally, we give some conclusions in Section 7.

## 2. Some Cases of Cryptojacking

### 2.1. Cryptojacking on Websites

Websites of different kinds have been victims of strong cryptojacking-type malicious software actions; some famous cases are: In early 2018, the online video-sharing platform YouTube was illegally compromised where the CoinHive miner ran on its ads [20,21], and the Russian Nuclear Weapon Research Center [22]

### 2.2. Cryptojacking with Advanced Techniques

Other attacks have used advanced techniques in the spread of cryptojacking; an example of this was when the botnet called vollgar attacked all Microsoft SQL (MSSQL) databases servers to take control of administrative accounts and inject malicious miners into said servers  [23]. Another example of the use of these advanced techniques was presented with the Zoom video conferencing software. In this case, the attackers merged cryptojacking malware with the main zoom application and published it on different file-sharing platforms [24]. Similarly, this technique was used in the Nintendo Switch consoles [25]. Another use case of these advanced techniques was presented in MikroTik routers between July and August of the year 2018, where a cryptojacking campaign managed to compromise more than 200,000 MikroTik routers; these routers were primarily located in Brazil; in the same way, researchers observed that routers that were not MikroTik were also compromised [26]. Moreover, a recent study explores cryptojacking and its impact on Electric Vehicles [27].

In 2019, eight applications were detected and removed from the Microsoft Store in Windows 10. When the user installed and opened these applications, they secretly downloaded cryptojacking’s JavaScript code, which carried out mining tasks for the Monero cryptocurrency, notably affecting the user device performance [28,29].

In 2018, researchers from RedLock dedicated to computer security discovered that at least one unknown computer criminal broke into an Amazon cloud account associated with Tesla and used it to mine cryptocurrencies. In this attack, the Stratum mining protocol was used, and the true IP address of the mining pool was hidden and kept the CPU consumption low [30].

### 2.3. Cryptojacking in Industrial Control Systems or Critical Servers

The impact of cryptojacking has surpassed the borders of traditional websites, affecting industrial control systems and Critical Servers; some of those cases are: In January 2020, following a report on the bug bounty website www.hackerone.com (accessed on 1 November 2022), the US Department of Defense discovered that its government and military servers were affected by cryptojacking to mine the currency Monero illegally  [31,32]. In 2019, a Russian nuclear warhead facility employee was fined around $7000 for illegally mining bitcoin using the facility’s servers [33]

## 3. Related Works

The literature presents different techniques for the detection of cryptojacking on websites; however, these solutions present some limitations related to performance, transparency and effectiveness.

A hardware-based approach to cryptojacking detection is presented in  [13]; this method takes advantage of the Intel Processor Trace mechanism to collect control flow information at runtime from the web browser. This technique uses two optimization approaches based on library functionality and information gain to preprocess control flow information. It also takes advantage of a Recurrent Neural Network (RNN) for cryptojacking detection.

A method that performs a fingerprinting technique to detect possible malicious sites is presented in  [14], which is then characterized with an autoencoding algorithm that keeps the best information of the infection vestiges to maximize the classification power by means of a deep dense neural network.

A lightweight cryptojacking detection system that uses deep learning techniques to accurately detect the presence of unwarranted mining activity based on emerging WebAssembly (Wasm)-based cryptojacking malware in real-time is introduced at  [15]. This system employs an image-based classification technique to distinguish between benign web pages and those using Wasm. Specifically, the classifier implements a convolutional neural network (CNN) model.

A detection and control method for IoT botnets is presented in  [16]; it uses a deep learning model, and cryptojacking activities carried out by the bot. This method performs malicious attack detection by implementing a sparse autoencoder composed of an input layer, a hidden layer, and an output layer.

A method for detecting silent browser mining behavior is presented in  [17]; this method drives known malicious mining samples, extracts heap snapshots and stack code functions of a dynamically running browser, and performs automatic detection based on a recurrent neural network (RNN).

A method called CapJack to identify illicit bitcoin mining activity in a web browser using cutting-edge CapsNet technology is presented in  [18]. Deep learning framework CapsNet employs heuristics based on system behavior to detect malware effectively.

A detection method called CoinPolice is presented in  [19]; that method flips throttling against cryptojackers, artificially varying the browser’s CPU power to observe the presence of throttling. Based on a deep neural network classifier, coinPolice can detect hidden miners.

## 4. Methodology

**Figure 1:**
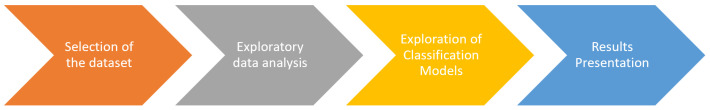
Methodology.

In this work, we follow a methodology, whose phases are shown in Figure 1 and described next.

1. Selection of the dataset.
2. Exploratory data analysis. In this phase, we calculate a correlation matrix and perform clustering of the dataset. Additionally, we perform feature selection proccess, dataset normalization and dataset splitting.
3. Exploration of Classification Models. In this phase, we reproduce the Sparse Autoencoder + Deep dense Neural Network model for Cryptojacking detection [14]. Additionally, we train and test different classifiers for Cryptojacking detection.
4. Results’ Presentation. We present the results of applying *K*-means clustering to the dataset, as well as the results of cross-validation and evaluation of the different classification models.

The development of these phases was carried out in a notebook in the Google Colaboratory (Google Colab) environment with the python programming language and libraries such as numpy, pandas, seaborn, matplotlib, tensorflow, keras and sklearn. The interested reader can see the source code here [34]. The different classification models were executed in the virtual machine offered by Google Colab, which has the following characteristics: 2.20 GHz Intel Xeon processor, 12G of RAM, and Ubuntu 18.04.6 LTS Operating system.

### 4.1. Selection of Dataset

We use the dataset that was collected in [14]. This dataset contains records of host-based and network-based features associated with websites. Moreover, each record is labeled whether a website is infected or not by cryptojacking. According to [14], the procedure to capture these samples includes three layers: the first of these is called fingerprinting, in which the sites that may contain signs of cryptomining are captured; after this, data based on the network is captured, which includes the information flows of the traffic that transits through the HTTP, HTTPS, and TCP protocols, and finally, the host-based data is captured, which consists of the tracking of the website once that interacts with the browser. The dataset used in this paper comprises 9292 benign sites and 3434 sites labeled and validated as cryptojacking infected. The host-based and network-based features that compose the dataset are indicated in Table 1  [14]. In addition to these features, the dataset presents an attribute called label, representing the class or classification of the sample, where 1 indicates a sample of a site labeled as cryptojacking infected and 0 as a benign site.

### 4.2. Exploratory Data Analysis

This section describes the data correlation and clustering, as well as the feature selection, splitting, and normalization process on the dataset.

#### 4.2.1. Correlation

**Figure 2:**
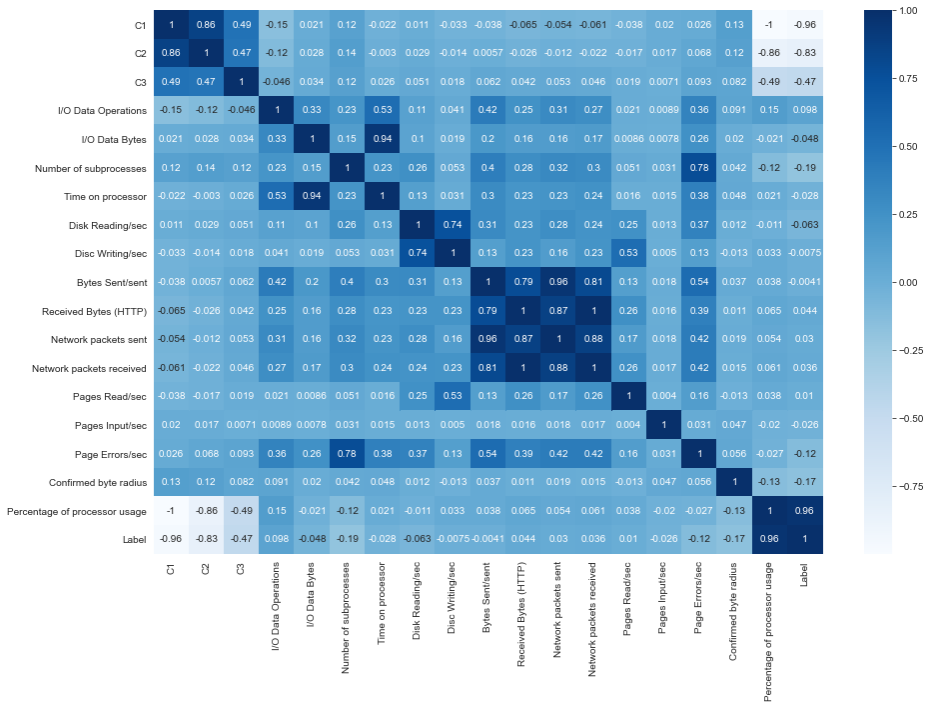
Feature correlation matrix.

We verify that the dataset does not present missing cells and duplicate rows. As expected, the dataset does not present these cases thanks to the fact that after its collection, its collectors went through a pre-processing process. To identify the existing correlation between the different features that make up the dataset and the classification class, we built a correlation matrix by employing the method corr of pandas. DataFrame with the default parameters (method = ‘pearson’, min_periods = 1, numeric_only = _NoDefault.no_default) (see Figure 2).

This matrix tells us that the feature “utility of the processor” is the one that presents the highest positive correlation with the classification of the website. On the other hand, the features C1, C2, C3, “Number of subprocesses”, “confirmed byte radius” and “Page Errors/sec” have, in their order, the highest negative correlation with website ranking.

#### 4.2.2. Clustering

**Figure 3:**
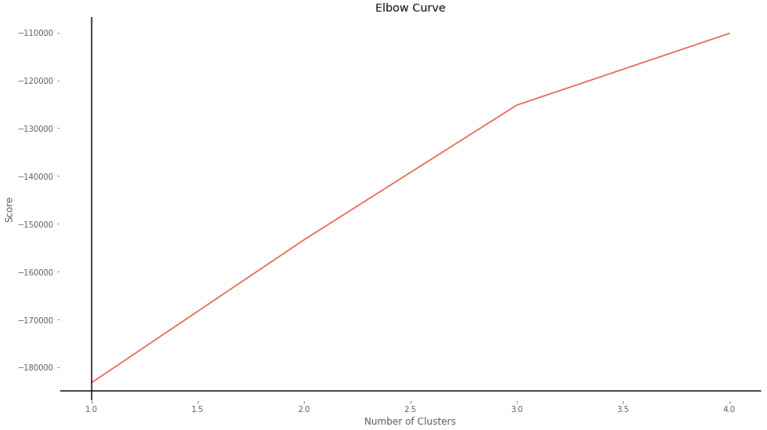
Elbow curve.

Due to the essence of the dataset, we know that its records are divided into two categories; the first is the records of sites infected with Cryptojacking (labeled with 1) and the records of benign sites (labeled with 0). To verify and analyze how well these records are grouped according to their features, we use the Unsupervised Learning algorithm *k*-Means Clustering, which groups the unlabeled dataset into different clusters. First, we used the elbow method to determine the number of clusters in the dataset. Figure 3 shows the elbow of the curve at three, but for the reason that we explained previously, we give two as the number of clusters parameter to the *k*-means algorithm.

Next, we cluster the dataset using the *k*-means algorithm with the parameters n_clusters = 2, random_state=0 and init= “*k*-means++”. “*k*-means++” is a method that chooses the first centroid to the location of a randomly selected data point and then chooses subsequent centroids of the remaining data points based on a probability proportional to the square of the distance from the nearest existing centroid of a given point. It helps in choosing the centroids to be as far away as possible, trying to cover the data space occupied since the initialization [35].

**Figure 4:**
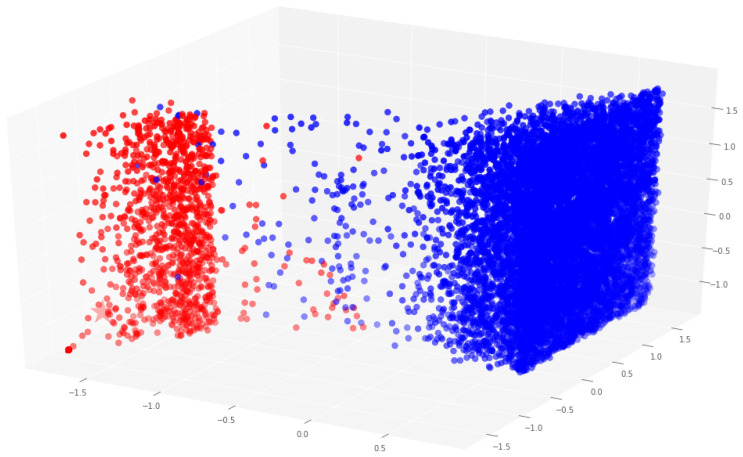
Clustering of dataset entries.

We apply the *k*-means algorithm to all the dataset entries and generate a figure to visualize the group assignment for each sample of the dataset group, only taking C1, C2, and C3 features. Figure 4 shows that the application of clustering to the dataset results in two well-defined groups corresponding to the samples of benign sites and infected websites.

#### 4.2.3. Feature Selection

Extracting relevant features from the raw data is paramount for Intrusion Detection System (IDS) classification [36]. For feature selection, first, we select the features with a *p*-value less than or equal to 0.05 and later apply statistical methods with the f_classif function and the univariate filtering function “Anova Scores” for 10 and 4 features. “Anova Score” fits a simple linear model between a single feature and the outcome, then the *p*-value for the whole model Ftest is returned [37]. In addition, an RFECV wrapper method was applied, which is a Feature ranking with recursive feature elimination and cross-validated selection of the best number of features [38]. The parameters used in the feature selection are in Table 2. For each of these filters, we extracted a subset of data that we used in the cross-validation.

#### 4.2.4. Split and Normalization of the Dataset

After exploring the dataset, we separate the features in a *X* set and the label category in a *Y* set; we then split the records of the dataset for training (80% of them) and testing (20% of them). For this partition, we use the parameters random_state=42 and stratify=y. The stratify parameter makes a split so that the proportion of values in the sample produced will be the same as the proportion of values provided to the parameter, stratify. Because the ranges for the different data features are very different, we apply the Standard Scaler to the training and testing datasets for the original dataset and the other datasets created as explained in Section 4.2.3.

### 4.3. Exploration of Classification Models

Having the dataset, we prepare a subset of training data and a subset of test data. Moreover, we applied normalization to the data. With the normalized data, we replicate the sparse autoencoder (SAE) + deep, dense neural network (DDNN) model, proposed in [14], to have a point of comparison for the proposed models. Later, some techniques are applied for the selection of features; after this, six reference models are defined for classification such as Logistic regression, Decision Tree, Random Forest, Gradient Boosting, *k*-Nearest Neighbor, and XGBoost. With these reference models, the different datasets obtained in the selection of features are tested, performing Cross-Validation for each of these sets. Finally, the performance of the classification models is measured using precision, recall, and F1-score.

#### 4.3.1. Sparse Autoencoder + Deep dense Neural Network

According to the results shown in [14], the Sparse Autoencoder + Deep dense Neural Network model is an state-of-the-art model for cryptojacking detection. We refer the reader to [14] to see a deep comparison between this model and other proposed models. In this paper, we reproduce this model to validate the results obtained with the corresponding dataset and thus have a point of comparison with the other models explored in this work.

#### 4.3.2. Classification Models

In this work, we consider the following classification models.

- Logistic regression or LR is a standard probabilistic statistical classification model widely used in different disciplines, such as computer vision, and marketing, among others [39].
- A Decision Tree is a hierarchical structure built using a data set’s features (independent variables). In a decision tree, each node is partitioned according to a measure associated with a subset of features [40]. This algorithm repeatedly divides the data set according to a criterion that seeks to maximize the separation of the data, resulting in a tree-like structure [41].
- A random forest is a collection of decision trees associated with a set of bootstrap samples generated from the original data set. The nodes are partitioned based on the entropy or Gini index of a selected subset of the features [40].
- Gradient Boosting is a widely used machine learning algorithm due to its efficiency, accuracy, and interpretability [42]. This algorithm achieves state-of-the-art performance in many machine learning tasks, such as multi-class classification [43], click prediction [44] and learning to rank [45].
- *k*-Nearest Neighbor classifier, unlike other methods, uses the data directly for classification, without first building a model [46,47]. One of the advantages of the *k*-nearest neighbors algorithm over other algorithms is the fact that the neighbors can provide an explanation for the classification result [48].
- XGBoost is a scalable ensemble machine learning and gradient boosting technique focusing on performance and speed. This technique allows for solving problems of ranking, classification, and regression [49].

We instantiate and train the classification models: Logistic Regression, Decision Tree, Random Forest, Gradient Boosting, *k*-Nearest Neighbor, and XGBoost. Table 3 shows the parameters used in these algorithms. With the different models, cross-validation is performed with the different datasets, and the mean and standard deviation of the accuracy are calculated.

## 5. Results

### 5.1. *K*-Means Clustering

Once the clustering model has been trained, we take the dataset and look for which element is the closest to the centroid of each of the two groups, these elements will be taken as the representatives of each of the groups. For each of these representatives, we verify the values of their features and category (Table 4 ). We identify that the category for each representative is different (benign/infected), and the values of some features are distant from each other.

With the clustering model created and trained, we apply this clustering model to the test dataset, obtaining the two clusters; with the help of the representative elements of each cluster, we identify which of the clusters corresponds to the benign sites and which to the infected ones. With this information, the cluster assigned to each element of the test dataset and the set of labels (y) corresponding to the test dataset, we calculate a classification report where we assume that the cluster assignment to each sample of the dataset corresponds to classification between the benign site and infected site. In this report, we obtain an accuracy = 0.9902 and precision = 0.988201.

### 5.2. Feature Selection

Table 5 presents the results of applying the methods for selecting features. It shows the method used and its selected features. We obtain 15 and 12 features with *p*-value and “RFECV wrapper” methods, respectively.

### 5.3. Cross-Validation

For the cross-validation and evaluation of the models, we employed some datasets, which are described in Table 6 and used in Table 7 and Table 8.

The results of the Cross-Validation of the different classification models with each of the datasets are presented in Table 7.

### 5.4. Model Selection and Evaluation

Table 8 shows the main metrics obtained with the classification models.

**Figure 5:**
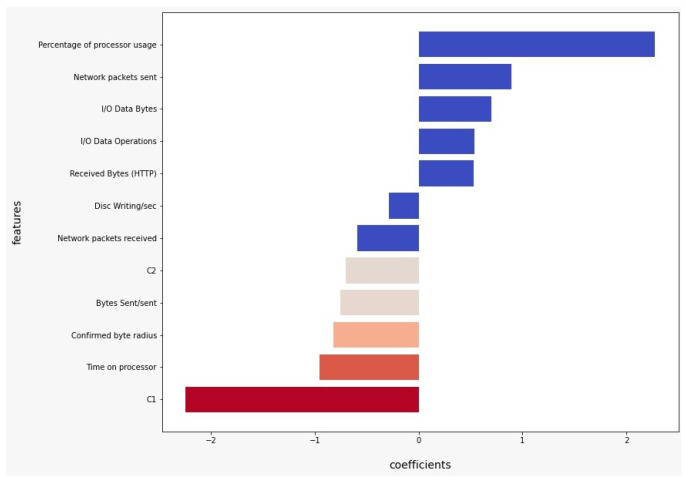
Feature importance logistic regression model.

**Figure 6:**
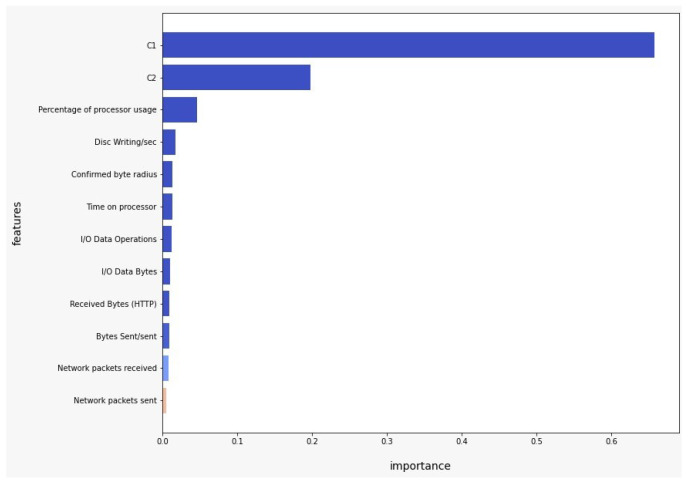
Feature importance XGBoost Model.

We take the simple model Logistic Regression and the advanced model XGBoost as a reference and search for the features with the most importance in the classification process. As observed in Figure 5, positive coefficients indicate that the event (malign) is more likely at that level of the predictor than at the reference level. Negative coefficients indicate that the event (malign) is less likely at that level of the predictor than at the reference level. It can be observed that the features’ Percentage of processor usage or Network packets sent indicate that the event is more likely, while the variables C1 or Confirmed byte radius suggest that the event is much less likely. As observed in Figure 6, we can notice that the variables C1, C2, Percentage of processor usage, and Disc Writing/sec are the most important in the classification process of XGBoost model.

## 6. Integration Scenario

Taking the ideas described in [50,51,52], we can think of implementing a hybrid, lightweight, usable, privacy-preserving mechanism added to a web browser for blocking websites that potentially may be infected by Cryptojacking. The envisioned approach exploits the blacklisting technique (widely used in this field) and a machine learning classifier to classify websites as benign or malign. The output from the classifier allows for updating the blacklists used to filter/block blacklisted websites. Additionally, this approach can be enhanced by introducing an ML-Based model to detect JavaScript malicious code inserted in websites or content shared with the user. To improve the usability of the mechanism, live alerts may be generated for the users for providing them with a comprehensive awareness and full control of potential cryptojacking threats.

## 7. Conclusions

In this work, we explored six Machine Learning models for Detecting cryptojacking on Websites. Our exploration started with a simple model as Logistic Regression, and then moved to more advanced algorithms in terms of tabular data classification, such as XGBoost, Decision Trees, Random Forest, Gradient Boosting, and *k*-Nearest Neighbor models. Furthermore, various feature selection methods were used, such as those based on statistical methods, e.g., Test Anova, and other methods called Wrappers, in order not only to reduce the complexity of the built models but also to know the features with greater predictive power.

From our results, we observed the following.

1. With 12 of the 18 features obtained with the RFECV method, an accuracy similar to that of other works [14,15,16,17,53] based on Deep Learning techniques was reached. Even, as observed in Table 7, with a dataset of only 4 features, an accuracy of 99.11% was obtained using Logistic Regression, and an accuracy of 99.13 was obtained with *k*-Nearest Neighbor.
2. The most relevant features in the case of Logistic Regression were C1, Percentage of processor usage, I/O Data Bytes and Network packets sent, while the most important features in the case of XGBoost were Percentage of processor usage, Network packets sent, Time on processor and C1.

We conclude that by using simple models such as Logistic Regression, Decision Tree, Random Forest, Gradient Boosting and *k*-Nearest Neighbor models, we can build ML-based classification components with a success rate similar to or greater than that of advanced algorithms such as XGBoost and even those of other works based on Deep Learning. Additionally, the simplicity of these models help the evaluator interpret the results and know the inner-working of these models in comparison with other advanced models based on Deep Learning (which are regarded as black boxes).

## Figures and Tables

**Table 1:** Host-based and Network-based Features.

Host-Based	
**Feature**	**Description**
C1	Time in C1 is a subset of the total processor idle time
C2	Time at C2 is a state of lower energy and higher output latency than Time at C1
C3	Time at C3 is a lower energy state and higher output latency than Time at C2
I/O Data Operations	Speed at which the process is issuing read and write I/O operations
I/O Data Bytes	Speed at which the process is reading and writing bytes in I/O operations
Number of subprocesses	Number of sub-processes that are currently active in a parent process
Time on processor	The total time, in seconds, that a process has been running
Disk Reading/sec	Speed of disk reading operations
Disc Writing/sec	Speed of writing operations to disk
Confirmed byte radius	The ratio of Memory/Bytes committed and Memory/Confirmation limit
Percentage of processor usage	Elapsed time on the processor when an active thread is running
Pages Read/sec	Speed rate at which the disk was read in order to resolve hard page errors
Pages Input/sec	Speed at which pages are written to disk to free up space in physical memory
Page Errors/sec	This is the average number of pages with faults per second
Bytes Sent	The rate at which bytes leave the browser’s HTTP requests
Received Bytes (HTTP)	Speed of bytes arriving to the browser’s HTTP responses
Network packets sent	Speed of sending packets in the TCP protocol
Network packets received	Packet reception speed over the TCP protocol

**Table 2:** Parameters used in the feature selection methods.

Method	Paramethers
*p*-value	*p*-value <= 0.05
Anova scores	n_splits=5, n_repeats=5, random_state=12345, score_func = f_classif, k= 5/10
RFECV wrapper	estimator = LogisticRegression, step = 1, cv = StratifiedKFold (n_splits = 5, random_state = 12345, shuffle = True), min_features_to_select = 1, scoring = ‘f1’, verbose = 2, n_jobs = −1

**Table 3:** Models’ parameters.

Model	Parameters
Logistic regression	default
Decision Tree	criterion = ‘gini’, max_depth = 5, min_samples_leaf = 20, random_state = 12345
Random Forest	n_estimators = 25
Gradient Boosting	n_estimators = 20, learning_rate = 0.5, max_features = 2, max_depth = 2, random_state = 0
*k*-Nearest Neighbor	n_neighbors = 36, p = 1 (manhattan_distance)
XGBoost	max_bin = 255

**Table 4:** Values of the features of representatives of clusters.

Feature	Cluster 1 (y = 0)	Cluster 2 (y = 1)
C1	82.2145	8.2148
C2	73.1083	25.4516
C3	3.9767	6.4803
I/O Data Operations	32.7094	18.8409
I/O Data Bytes	121124.1414	39403.4743
Number of subprocesses	30	31
Time on processor	0.4967	0.1733
Disk Reading/sec	16.6267	4.9044
Disc Writing/sec	1.3781	0
Bytes Sent/sent	915.6518	210.0906
Received Bytes (HTTP)	12258.8161	3844.3897
Network packets sent	6.1794	1.6865
Network packets received	10.1138	3.6172
Pages Read/sec	0.6446	0.0665
Pages Input/sec	0	0
Page Errors/sec	13351.0499	1633.1676
Confirmed byte radius	28.9634	27.1788
Percentage of processor usage	17.7854	91.7852

**Table 5:** Selected features.

Method	Selected Features
*p*-value	C1, C2, C3,I/O Data Operations, I/O Data Bytes, Number of subprocesses, Time on processor, Disk Reading/sec, Received Bytes (HTTP), Network packets sent, Network packets received, Pages Input/sec, Page Errors/sec, Confirmed byte radius, Percentage of processor usage
anova scores 4 features	Percentage of processor usage, C1, C2, C3
anova scores 10 features	Percentage of processor usage, C1, C2, C3, Number of subprocesses, Confirmed byte radius, Page Errors/sec, I/O Data Operations, Disk Reading/sec, I/O Data Bytes
RFECV wrapper	Percentage of processor usage, Network packets sent, I/O Data Bytes, I/O Data Operations, Received Bytes (HTTP), Disc Writing/sec, Network packets received, C2, Bytes Sent/sent, Confirmed byte radius, Time on processor, C1

**Table 6:** Datasets’ description.

Dataset Name	Description
All features dataset	complete dataset
15 features dataset	dataset of 15 features selected with *p*-value
4 features dataset	dataset of 4 features selected with anova Scores method
10 features dataset	dataset of 10 features selected with anova Scores method
12 features dataset	dataset of 12 features selected with RFECV wrapper method

**Table 7:** Cross-validation models.

Logistic Regression		
**Dataset**	**Mean Accuracy**	**Standard Deviation Accuracy**
All features dataset	0.9917	0.0020
15 features dataset	0.9911	0.0020
4 features dataset	0.9911	0.0023
10 features dataset	0.9912	0.0021
12 features dataset	0.9919	0.0018
**Decision Tree**		
All features dataset	0.9914	0.0018
15 features dataset	0.9921	0.0022
4 features dataset	0.991	0.0018
10 features dataset	0.991	0.0018
12 features dataset	0.9915	0.0020
**Random Forest**		
All features dataset	0.9944	0.0015
15 features dataset	0.9944	0.0014
4 features dataset	0.9906	0.0017
10 features dataset	0.9943	0.0015
12 features dataset	0.9947	0.0013
**Gradient Boosting**		
All features dataset	0.9926	0.0018
15 features dataset	0.9919	0.0019
4 features dataset	0.9905	0.0022
10 features dataset	0.9919	0.0020
12 features dataset	0.9932	0.0016
**k-Nearest Neighbor**		
All features dataset	0.9878	0.0018
15 features dataset	0.9878	0.0020
4 features dataset	0.9913	0.0021
10 features dataset	0.9891	0.0019
12 features dataset	0.9889	0.0019
**XGBoost**		
All features dataset	0.9947	0.0016
15 features dataset	0.9946	0.0016
4 features dataset	0.9908	0.0018
10 features dataset	0.9942	0.0017
12 features dataset	0.9950	0.0017

**Table 8:** Results of evaluating the models.

SAE + DDNN [14]							
		benign			malign		
Dataset	Accuracy	Precision	Recall	F1-score	Precision	Recall	F1-score
All features dataset	0.9893	0.9941	0.9911	0.9926	0.9823	0.9712	0.9891
Logistic regression							
		benign			malign		
Dataset	Accuracy	Precision	Recall	F1-score	Precision	Recall	F1-score
All features dataset	0.9937	0.9957	0.9957	0.9957	0.9884	0.9884	0.9884
15 features dataset	0.9937	0.9957	0.9957	0.9957	0.9884	0.9884	0.9884
4 features dataset	0.9918	0.9946	0.9941	0.9944	0.984	0.9854	0.9847
10 features dataset	0.9925	0.9957	0.9941	0.9949	0.9841	0.9884	0.9862
12 features dataset	0.9941	0.9957	0.9962	0.996	0.9898	0.884	0.9891
Decision Tree							
		benign			malign		
Dataset	Accuracy	Precision	Recall	F1-score	Precision	Recall	F1-score
All features dataset	0.9902	0.9904	0.9962	0.9933	0.9896	0.9738	0.9817
15 features dataset	0.9918	0.993	0.9957	0.9944	0.9883	0.9811	0.9847
4 features dataset	0.9906	0.9925	0.9946	0.9936	0.9854	0.9796	0.9825
10 features dataset	0.9906	0.9946	0.9925	0.9935	0.9797	0.9854	0.9826
12 features dataset	0.9918	0.993	0.9957	0.9944	0.9883	0.9811	0.9847
Random Forest							
		benign			malign		
Dataset	Accuracy	Precision	Recall	F1-score	Precision	Recall	F1-score
All features dataset	0.9957	0.9957	0.9984	0.997	0.9956	0.9884	0.992
15 features dataset	0.9953	0.9962	0.9973	0.9968	0.9927	0.9898	0.9913
4 features dataset	0.9906	0.9925	0.9946	0.9936	0.9854	0.9796	0.9825
10 features dataset	0.9949	0.9952	0.9978	0.9965	0.9941	0.9869	0.9905
12 features dataset	0.9949	0.9952	0.9978	0.9965	0.9941	0.9869	0.9905
Gradient Boosting							
		benign			malign		
Dataset	Accuracy	Precision	Recall	F1-score	Precision	Recall	F1-score
All features dataset	0.9937	0.9957	0.9957	0.9957	0.9884	0.9884	0.9884
15 features dataset	0.9929	0.9952	0.9952	0.9952	0.9869	0.9869	0.9869
4 features dataset	0.991	0.9935	0.9941	0.9938	0.984	0.9825	0.9832
10 features dataset	0.9925	0.9946	0.9952	0.9949	0.9869	0.9854	0.9862
12 features dataset	0.9929	0.9946	0.9957	0.9952	0.9883	0.9854	0.9869
*k*-Nearest Neighbor							
		benign			malign		
Dataset	Accuracy	Precision	Recall	F1-score	Precision	Recall	F1-score
All features dataset	0.9906	0.9904	0.9968	0.9936	0.9911	0.9738	0.9824
15 features dataset	0.9906	0.9909	0.9962	0.9936	0.9897	0.9753	0.9824
4 features dataset	0.9921	0.9946	0.9946	0.9946	0.9854	0.9854	0.9854
10 features dataset	0.9894	0.9909	0.9946	0.9928	0.9853	0.9753	0.9802
12 features dataset	0.9902	0.992	0.9946	0.9933	0.9853	0.9782	0.9817
XGBoost							
		benign			malign		
Dataset	Accuracy	Precision	Recall	F1-score	Precision	Recall	F1-score
All features dataset	0.9965	0.9973	0.9978	0.9976	0.9942	0.9927	0.9934
15 features dataset	0.9965	0.9973	0.9978	0.9976	0.9942	0.9927	0.9934
4 features dataset	0.9910	0.9925	0.9952	0.9938	0.9868	0.9796	0.9832
10 features dataset	0.9965	0.9973	0.9978	0.9976	0.9942	0.9927	0.9934
12 features dataset	0.9965	0.9973	0.9978	0.9976	0.9942	0.9927	0.9934

## Data Availability

The data presented in this study are available on request from the corresponding author.
